# Assessment of the nutritional status of 6–36-month-old infants in Xinjiang and analysis of the influencing factors

**DOI:** 10.1038/s41598-020-78042-6

**Published:** 2020-12-03

**Authors:** Meng Wei, Jing Li, Mei Yan, Maimaiti Rena, Hui Zhang, Ju Dong

**Affiliations:** 1 The First Affiliated Hospital of Xinjiang Medical University, Urumqi, 830054 China; 2 Pediatric Center, The First Affiliated Hospital of Xinjiang Medical University, Urumqi, 830054 China; 3 Children’s Health Department of Health Management Hospital, The First Affiliated Hospital of Xinjiang Medical University, Urumqi, 830054 China; 4 Department of Maternal and Child Health and Community Health, Disease Prevention and Control Center of Xinjiang Production and Construction Corps, Urumqi, 830001 China; 5 Outpatient and Emergency Departments of Pediatrics, The First Affiliated Hospital of Xinjiang Medical University, Urumqi, 830054 China

**Keywords:** Health care, Risk factors

## Abstract

The health of infants is not only related to family happiness, but also to the future and development of the country. Therefore, it is still a very important public health problem to pay attention to the nutritional health level of infants. This article explores the nutritional health levels of infants and reveals the related risk factors. Stratified and multi-stage cluster sampling was used to select 3949 infants and young children in Xinjiang for the study. Survey staff conducted face-to-face questionnaire surveys to investigate their demographic characteristics, complementary food feeding, and related risk factors affecting their nutritional status. Study results showed that compared with the Han nationality, the Uygur and other nationalities were independent risk factors for malnutrition, as odds ratio (OR) values were 2.456 and 1.747, respectively (*P* < 0.05). When the feeders were not maternal, and their educational background was junior high school or below, OR values were 2.122 and 1.810, respectively (*P* <  0.05). The scores for non-breastfeeding and feeding behaviors were independent risk factors, and OR values were 1.983 and 2.709, respectively (*P* <  0.05). When infant minimum dietary diversity, minimum meal frequency, and minimum acceptable diet were unqualified, these indices were independent risk factors, and OR values were 2.281, 2.315, and 1.865, respectively (*P* <  0.05). The healthy growth of Han infants is better than that of other ethnic groups, which may be related to varying living environments, customs, social/economic development levels, educational levels, and other factors. In the future, the focus of our work should be to cooperate with the superior health organization to strengthen and improve the nutritional health level of infants.

## Introduction

The health of infants and young children is not only related to family happiness, but also to the future and development of the country. Strengthening and developing child medical services is an important part of China's health construction and public health development. It is of great significance to ensure that all residents experience improvements in their livelihood and health quality; however, infants and young children are in a critical period of growth and development. Good nutrition is essential for their growth and health^1^. The nutritional status of infants and young children has always been a field of great concern to the international community^2^. Xinjiang is located in the northwest of China, with a large number of ethnic groups, and its economic and cultural levels are relatively backward compared with those in the central and southeast regions. Therefore, this sampling survey focuses on infants and young children of all ethnic groups in Xinjiang, mainly using face-to-face interviews in the form of questionnaires, to investigate their nutritional health levels in order to determine the relevant risk factors for the future and provide a scientific basis for the development of targeted nutritional interventions.

## Materials and methods

### Objective

In this survey, from July 2017 to August 2018, we selected Hetian, Kashi, Aksu, Changji Hui Autonomous Prefecture, and Yili Prefecture in southern Xinjiang, and we further selected Moyu, Hetian, Shufu, Yuepuhu, Kashi, Jimusar, and Hutubi counties in Hetian, Kashi, and Changji Hui Autonomous Prefecture by a stratified multi-stage cluster sampling method. 2 towns and 5 villages were selected from each county, and 6–36-month-old infants were investigated. A total of 3949 infants and young children were included in this study.

### Epidemiological investigation methods

#### Questionnaire survey

A cross-sectional study was conducted using a questionnaire survey. The questionnaire was repeatedly discussed and modified by the expert group. The content of the document included the general situation, needs, and relevant details of the infants and young children. Before administering the survey, the survey staff was trained in a unified way, and the survey was conducted using a unified guidance language. At the same time as the one-on-one inquiry, the qualified investigators and translators completed the questionnaire. This study was approved by the ethics committee of the First Affiliated Hospital of Xinjiang Medical University (K201701-01). This study complies with the international declaration of Helsinki, the ethical examination and Approval Measures for biomedical research involving human beings (Implementation), as well as the requirements of relevant laws and regulations. Informed consent was obtained from all subjects or, if subjects are under 18, from a parent and/or legal guardian.

#### Definition of indicators and outcome indicators

In this study, we used the instruments we have equipped to uniformly measure and record the body length and weight of infants and young children with standard measurement methods. Physical measurement assessed height and weight requirements using standard measurement tools (accuracy: height/0.01 kg; weight/0.1 cm).

The qualified rate of minimum dietary diversity (MDD) was defined as follows: Within 24 h, there were 4 or more kinds of complementary foods for 6–23-month-old infants. The types of food were divided into vegetables (fruits), eggs, meat, fish and shrimp, beans and bean products, milk and milk products, and cereals and potatoes^3^. The qualified rate of the minimum meal frequency (MMF) was defined as: the proportion of 6–23-month-old infants who added complementary foods to meet the minimum feeding times during the past 24 h—2 times for 6–8-month-old infants who were breastfed, 3 times for 9–23-month-old infants who were breastfed, 4 times for 6–23-month-old infants who were not breastfed ^3^. The qualified rate of the minimum acceptable diet (MAD) was defined as follows: the proportion of the children aged 6–23 months who added supplementary food to reach the lowest dietary frequency in the past 24 h^3^. Breastfed infants’ MAD refers to meeting both MDD and MMF, while non-breastfed infants’ MAD refers to receiving at least 2 milk feedings and meeting both MDD (excluding milk) and MMF. Breastfeeding was defined as follows: Breast milk was included in the food intake of infants within 24 h before the survey. Non-breastfeeding was defined as follows: Breast milk was not included in the food consumed by infants within 24 h before the survey.

The feeding behavior score of the feeders was based on the following 14 topics: preparing food for the children alone, paying attention to the age needs during food preparation, having a fixed feeding position, etc., and each question was divided into five grades with a scale of 1–5 points.

The Z-score method was used to evaluate the nutritional status of infants and young children, and height for age Z score (HAZ)^4^, weight for age Z score (WAZ)^4^, weight for height Z score (WHZ)^4^ were calculated based on the reference values of gender and age-specific height and weight from 2006 WHO standards. The calculation formula for Z value is as follows:

$$ {\text{Z}} = \frac{{\left( {{\text{physical}}\, {\text{measurement}}\, {\text{value}} - {\text{median}}\, {\text{of}}\, {\text{WHO}}\, {\text{standard}}\, {\text{for}}\, {\text{the}}\, {\text{same}}\, {\text{age}}\, {\text{and}}\, {\text{same}}\, {\text{sex}}} \right)}}{{{\text{standard}}\, {\text{deviation}}\, {\text{of}}\, {\text{WHO}}\, {\text{s}}\tan {\text{dard}}}}. $$

The z-values of age-specific height, age-specific weight, and height-specific weight lower than 2 standard deviations were growth retardation, low weight, and emaciation, respectively, and any 1 or more of growth retardation, low weight, or emaciation in this study was considered malnutrition^5^.

### Statistical analysis

All data collected in this study were stored in epidata3.1 and entered by two people. SPSS 17.0 was used for data analysis. The mean ± standard deviation was used to describe the measurement data in the results of this study. ANOVA was used to compare the mean among multiple groups, and the Bonferroni method was used to compare the two groups. We used 2 tests to classify the data. The factors influencing infant malnutrition were analyzed by unconditional single factor and multi-factor logistic regression analyses. In this study, α = 0.05 was taken as the statistical test level.

### Ethical approval

The study design was approved by the appropriate ethics review board. The subjects are under 18, from a parent and/or legal guardian.

### Informed consent

Informed consent was obtained from all subjects or, if subjects are under 18, from a parent and/or legal guardian.

## Results

### General information of the subjects

This study found that the family and child populations of Uygur, Hui, and other nationalities were higher than that of Han (*P* <  0.05). There was no difference in the sex of infants, the composition of each age group, and the proportion of registered permanent residences among all ethnic groups (*P* > 0.05). In the living areas, we found that the Han and Hui people mainly live in northern Xinjiang, while the Uygur and other ethnic minorities mainly live in southern Xinjiang. Regardless of the ethnic group being assessed, the proportion of children who were fed by their mothers was the highest. Compared with other ethnic groups, the proportion of Han infants who were the first children was the highest, and the educational background of Han feeders was also higher than that of other ethnic groups (*P* <  0.05). However, the results showed that the proportion of breast-feeding Han mothers was lower than that of other nationalities (*P* <  0.05). The score, height, and weight of the feeders in these ethnic groups were higher than those in other ethnic groups (*P* <  0.05). The specific data are shown in the table below, and the details are shown in Table 1.

**Table 1:** Demographic characteristics of subjects.

Demographic characteristics		Han	Uygur		Hui		Other nations		F/χ^2^	*P*
Family size		4.23 ± 0.62	5.32 ± 0.79		5.42 ± 1.14		5.17 ± 0.83		589.299	< 0.001
Number of children in the family		1.58 ± 0.37	2.14 ± 0.50		2.18 ± 0.46		2.03 ± 0.41		529.357	< 0.001
Sex	Male	873 (51.44%)	476 (51.80%)		326 (52.24%)		374 (52.75%)		0.38	0.944
	Female	824 (48.56%)	443 (48.20%)		298 (47.76%)		335 (47.25%)			
Registered permanent residence	Rural	731 (43.08%)	417 (45.38%)		264 (42.31%)		328 (46.26%)		3.484	0.323
	Urban	966 (56.92%)	502 (54.62%)		360 (57.69%)		381 (53.74%)			
Living area	Southern Xinjiang	312 (18.39%)	683 (74.32%)		146 (23.40%)		439 (61.92%)		994.868	< 0.001
	Northern Xinjiang	1385 (81.61%)	236 (25.68%)		478 (76.60%)		270 (38.08%)			
Infant age	6–12 months	209 (12.32%)	105 (11.42%)		58 (9.29%)		98 (13.82%)		14.703	0.258
	13–18 months	586 (34.53%)	330 (35.91%)		215 (34.46%)		240 (33.85%)			
	19–24 months	618 (36.42%)	315 (34.28%)		229 (36.70%)		241 (33.99%)			
	25–30 months	151 (8.90%)	83 (9.03%)		69 (11.06%)		78 (11.01%)			
	31–36 months	133 (7.83%)	86 (9.36%)		53 (8.49%)		52 (7.33%)			
Infant feeding	Mother	952 (56.10%)	562 (61.15%)		325 (52.08%)		377 (53.17%)		31.978	< 0.001
	Father	190 (11.20%)	85 (9.25%)		70 (11.22%)		79 (11.15%)			
	Grandmother or grandfather	301 (17.74%)	109 (11.86%)		112 (17.95%)		115 (16.22%)			
	Others	254 (14.96%)	163 (17.74%)		117 (18.75%)		138 (19.46%)			
First child or not	Yes	1136 (66.94%)	453 (49.29%)		364 (58.33%)		397 (55.99%)		82.707	< 0.001
	No	561 (33.06%)	466 (50.71%)		260 (41.67%)		312 (44.01%)			
Educational background of feeder	Junior high school and below	781 (46.02%)	611 (66.49%)		397 (63.63%)		486 (68.55%)		194.053	< 0.001
	High school/technical secondary school	723 (42.60%)	279 (30.36%)		174 (27.88%)		196 (27.64%)			
	Junior college or above	193 (11.38%)	29 (3.15%)		53 (8.49%)		27 (3.81%)			
Breastfeeding	Yes	668 (39.36%)	527 (57.34%)		269 (43.11%)		366 (51.62%)		88.549	< 0.001
	No	1029 (60.64%)	392 (42.66%)		355 (56.89%)		343 (48.38%)			
Feeding behavior score of feeders		53.85 ± 6.97	35.26 ± 5.14*		42.52 ± 4.36*		38.87 ± 5.25*		761.57	< 0.001
Infant height (CM)		77.85 ± 8.17	73.33 ± 6.83*		75.29 ± 8.42*		74.69 ± 7.25*		75.308	< 0.001
Infant weight (kg)		10.06 ± 2.15	9.31 ± 2.21*		9.69 ± 2.32*		9.46 ± 2.25*		26.821	< 0.001

*Represents the comparison with Han nationality (*P* <  0.05).

### Comparison of complementary feeding rate among Han, Uygur, Hui, and other nationalities in Xinjiang

The results of this study showed that the MDD, MMF, and MAD rates of most age groups were lower than those of the Han nationality (*P* <  0.05). In the age group of 6–12 months, the MDD qualification rate of the Han nationality was the lowest (*P* <  0.05); the MDD qualification rate of each nationality was 25–30 and 31–36 months, and there was no difference (*P* > 0.05); the MMF qualification rate of each nationality was 6–12, 19–24, 25–30, and 31–36 months, and there was no difference (*P* > 0.05); the MAD qualification rate of each nationality was 6–12 and 25–30 months, and there was no difference (*P* > 0.05). The specific data are shown in the table below, and the details are shown in Table 2.

**Table 2:** Comparison of complementary feeding rates among Han, Uygur, Hui, and other nationalities in Xinjiang.

Index	Han	Uygur	Hui	Other nations	χ^2^	*P*
MDD pass rate	1295/1697 (76.31%)	637/919 (69.31%)	417/624 (66.83%)	454/709 (64.03%)	46.485	< 0.001
6–12 months	86/209 (41.15%)	61/105 (58.10%)	30/58 (51.72%)	53/98 (54.08%)	9.816	0.020
13–18 months	467/586 (79.69%)	218/330 (66.06%)	139/215 (64.65%)	142/240 (59.17%)	45.26	< 0.001
19–24 months	496/618 (80.26%)	227/315 (72.06%)	149/229 (65.07%)	152/241 (63.07%)	36.02	< 0.001
25–30 months	125/151 (82.78%)	65/83 (78.31%)	56/69 (81.16%)	64/78 (82.05%)	0.736	0.865
31–36 months	121/133 (90.98%)	66/86 (76.74%)	43/53 (81.13%)	43/52 (82.69%)	8.675	0.034
MMF pass rate	1332/1697 (78.49%)	681/919 (74.10%)	475/624 (76.12%)	519/709 (73.20%)	10.637	0.014
6–12 months	21/209 (10.05%)	15/105 (14.29%)	9/58 (15.52%)	16/98 (16.33%)	3.052	0.384
13–18 months	512/586 (87.37%)	253/330 (76.67%)	169/215 (78.60%)	186/240 (77.50%)	22.403	< 0.001
19–24 months	523/618 (84.63%)	256/315 (81.27%)	184/229 (80.35%)	197/241 (81.74%)	3.091	0.378
25–30 months	145/151 (96.03%)	76/83 (91.57%)	64/69 (92.75%)	73/78 (93.59%)	2.171	0.538
31–36 months	131/133 (98.50%)	81/86 (94.19%)	49/53 (92.45%)	47/52 (90.38%)	6.674	0.083
MAD pass rate	1136/1697 (66.94%)	541/919 (58.87%)	381/624 (61.06%)	409/709 (57.69%)	27.008	< 0.001
6–12 months	9/209 (4.31%)	8/105 (7.62%)	5/58 (8.62%)	8/98 (8.16%)	2.783	0.426
13–18 months	403/586 (68.77%)	191/330 (57.88%)	136/215 (63.26%)	149/240 (62.08%)	11.600	0.009
19–24 months	482/618 (77.99%)	217/315 (68.89%)	146/229 (63.76%)	150/241 (62.24%)	30.126	< 0.001
25–30 months	124/151 (82.12%)	61/83 (73.49%)	52/69 (75.36%)	61/78 (78.21%)	2.766	0.429
31–36 months	118/133 (88.72%)	64/86 (74.42%)	42/53 (79.25%)	41/52 (78.85%)	7.961	0.047

### Comparison of WAZ, HAZ, and WHZ of Han, Uygur, Hui, and other nationalities in Xinjiang

The results of this study show that for most age groups, the Han nationality is higher than other ethnic groups in WAZ, HAZ, and WHZ (*P* <  0.05), and the Uygur nationality is the lowest. However, there was no difference in WHZ among different nationalities in the age group of 25–30 (*P* > 0.05). The specific data is shown in the table below, and the details are shown in Table 3.

**Table 3:** Comparison of WAZ, HAZ, and WHZ mean of Han, Uygur, Hui, and other nationalities in Xinjiang.

Index		Han	Uygur	Hui	Other nations	*F*	*P*
WAZ	6–12 months	0.31 ± 0.52	0.11 ± 0.49*	0.27 ± 0.61	0.25 ± 0.32	3.405	0.009
	13–18 months	− 0.13 ± 0.33	− 0.36 ± 0.34*	− 0.18 ± 0.38	− 0.21 ± 0.36*	31.633	< 0.001
	19–24 months	− 0.35 ± 0.39	− 0.52 ± 0.45*	− 0.41 ± 0.33	− 0.40 ± 0.53	11.288	< 0.001
	25–30 months	− 0.43 ± 0.38	− 0.73 ± 0.56*	− 0.49 ± 0.37	− 0.61 ± 0.39*	9.875	< 0.001
	31–36 months	− 0.52 ± 0.34	− 0.82 ± 0.28*	− 0.61 ± 0.45	− 0.58 ± 0.37*	13.128	< 0.001
HAZ	6–12 months	− 0.16 ± 0.55	− 0.37 ± 0.42*	− 0.20 ± 0.24	− 0.21 ± 0.49	4.531	0.004
	13–18 months	− 0.45 ± 0.32	− 0.62 ± 0.39*	− 0.52 ± 0.23*	− 0.49 ± 0.30	19.846	< 0.001
	19–24 months	− 0.66 ± 0.47	− 0.85 ± 0.32*	− 0.78 ± 0.43*	− 0.74 ± 0.51	13.853	< 0.001
	25–30 months	− 0.79 ± 0.15	− 0.98 ± 0.26*	− 0.87 ± 0.42	− 0.89 ± 0.29*	9.162	< 0.001
	31–36 months	− 0.94 ± 0.81	− 1.24 ± 0.72*	− 1.01 ± 0.75	− 0.97 ± 0.66	2.967	0.032
WHZ	6–12 months	0.81 ± 0.93	0.53 ± 0.76*	0.68 ± 0.43	0.65 ± 0.41	3.394	0.018
	13–18 months	0.64 ± 0.30	0.45 ± 0.36*	0.53 ± 0.24*	0.50 ± 0.33*	29.745	< 0.001
	19–24 months	0.49 ± 0.36	0.32 ± 0.24*	0.44 ± 0.39	0.43 ± 0.41	16.283	< 0.001
	25–30 months	0.35 ± 0.27	0.30 ± 0.25*	0.31 ± 0.20	0.32 ± 0.21	0.927	0.428
	31–36 months	0.29 ± 0.24	0.18 ± 0.27*	0.23 ± 0.22	0.25 ± 0.32	3.198	0.024

*Represents the comparison with Han nationality (*P* <  0.05).

### Comparison of growth retardation, low weight, emaciation, and malnutrition among Han, Uygur, Hui, and other nationalities in Xinjiang

The results of this study showed that the incidence of growth retardation, low weight, wasting, and malnutrition in other nationalities were higher than that in the Han nationality (*P* <  0.05). Compared with other nationalities, the prevalence of malnutrition in the Uygur nationality was the highest, which could reach 30.25%. The specific data are shown in the table below, and the details are shown in Table 4.

**Table 4:** Comparison of the prevalence of stunting, low weight, wasting and malnutrition among Han, Uygur, Hui, and other nationalities in Xinjiang.

Index	Han	Uygur	Hui	Other nations	χ^2^	*P*
Growth retardation	247 (14.56%)	183 (19.91%)	112 (17.95%)	146 (20.59%)	18.732	< 0.001
Low body weight	111 (6.54%)	95 (10.34%)	56 (8.97%)	78 (11.00%)	17.976	< 0.001
Emaciation	76 (4.48%)	52 (5.66%)	28 (4.49%)	39 (5.50%)	334.099	< 0.001
Malnutrition	343 (20.21%)	278 (30.25%)	168 (26.92%)	199 (28.07%)	39.027	< 0.001

### Unconditional univariate logistic regression analysis of infant malnutrition

The results showed that compared with the Han nationality, Uighur, Hui, and other nationalities were risk factors, and the odds ratio (OR) values were 1.789, 1.121, and 1.365, respectively (*P* <  0.05). The rural area was a risk factor (OR 1.858) and North Xinjiang was a protection factor (OR 0.532) (*P* <  0.05). The risk of infant malnutrition was 2.483 times higher than that of the mother when the feeders were not the mothers. Junior high school education and non-breastfeeding were risk factors (OR 1.554 and OR 2.965, respectively) (*P* <  0.05). With feeding behavior scores ≤ 42, malnutrition was 1.221 times higher than 42. When MDD, MMF, and MAD were unqualified, OR values were 1.384, 2.164, and 1.793, respectively (*P* <  0.05). The specific data are shown in the table below, and the details are shown in Table 5.

**Table 5:** Single factor unconditional logistic regression analysis of infant malnutrition.

Variables	β	SE	Waldχ^2^	OR (95% CI)	*P*
**Nation**					
Han	–	–	–	1	–
Uygur	0.527	0.455	4.749	1.789 (1.229, 6.332)	0.002
Hui	0.423	0.212	3.713	1.121 (1.009, 4.965)	0.015
Other nations	0.517	0.442	4.369	1.365 (1.085, 5.023)	0.004
**Registered permanent residence**					
Rural	–	–	–	1	–
Urban	0.558	0.371	5.444	1.858 (1.325, 5.127)	< 0.001
**Living area**					
Southern Xinjiang	–	–	–	1	–
Northern Xinjiang	− 0.586	0.386	5.567	0.532 (0.186, 0.761)	< 0.001
**Infant feeding**					
Mother	–	–	–	1	–
The others	0.911	0.428	4.385	2.483 (1.714, 3.597)	< 0.001
**First child or not**					
Yes	–	–	–	1	–
No	0.392	0.391	4.214	1.103 (0.924, 3.652)	0.097
**Educational background of feeder**					
High school and above	–	–	–	1	–
Junior high school and below	0.441	0.336	4.126	1.554 (1.294, 4.317)	< 0.001
**Breastfeeding**					
Yes	–	–	–	1	–
No	1.087	0.354	3.827	2.965 (1.929, 4.559)	< 0.001
**Feeding behavior score of feeders**					
> 42	–	–	–	1	–
≤ 42	0.248	0.234	3.335	1.221 (1.111, 1.431)	0.003
**MDD**					
Qualified	–	–	–	1	–
Unqualified	0.324	0.295	2.328	1.384 (1.124, 1.821)	0.013
**MMF**					
Qualified	–	–	–	1	–
Unqualified	0.628	0.248	3.352	2.164 (1.469, 3.451)	< 0.001
**MAD**					
Qualified	–	–	–	1	–
Unqualified	0.486	0.431	3.985	1.793 (1.352, 4.128)	< 0.001

### Unconditional multivariate logistic regression analysis of infant malnutrition

After multiple factor analysis, it was found that compared with the Han nationality, the Uygur and other nationalities were independent risk factors (OR 2.456 and OR 1.747, respectively) (*P* <  0.05), and the rural area was also an independent risk factor (OR 2.452) (*P* <  0.05). The OR values were 2.122 and 1.810, respectively (*P* <  0.05) when the feeders were not the mothers and their educational background was junior high school or below. The OR values were 1.983 and 2.709, respectively (*P* < 0.05) when the scores for non-breastfeeding and feeding behaviors were less than 42. When the MDD, MMF, and MAD of infants were unqualified, these three indices were also independent risk factors, and their OR values were 2.281, 2.315, and 1.865, respectively (*P* <  0.05). The specific data are shown in the table below, and the details are shown in Table 6.

**Table 6:** The results of multiple factor unconditional logistic regression analysis on infant malnutrition.

Variables	β	SE	Waldχ^2^	OR (95% CI)	*P*
**Nation**					
Han	–	–	–	1	–
Uygur	0.948	0.425	4.336	2.456 (1.714, 3.597)	< 0.001
Hui	0.264	0.276	3.138	1.301 (1.051, 2.569)	0.018
Other nations	0.558	0.283	3.351	1.747 (1.339, 2.722)	0.004
**Registered permanent residence**					
Rural	–	–	–	1	–
Urban	0.897	0.211	2.548	2.452 (1.867, 3.455)	< 0.001
**Infant feeding**					
Mother	–	–	–	1	–
The others	0.752	0.295	3.475	2.122 (1.524, 3.185)	< 0.001
**Educational background of feeder**					
High school and above	–	–	–	1	–
Junior high school and below	0.594	0.357	4.125	1.810 (1.231, 2.832)	< 0.001
**Breastfeeding**					
Yes	–	–	–	1	–
No	0.685	0.306	3.518	1.983 (1.411, 3.448)	< 0.001
**Feeding behavior score of feeders**					
> 42	–	–	–	1	–
≤ 42	0.732	0.313	3.735	2.709 (1.402, 4.452)	< 0.001
**MDD**					
Qualified	–	–	–	1	–
Unqualified	0.556	0.328	3.437	2.281 (1.627, 3.343)	0.013
**MMF**					
Qualified	–	–	–	1	–
Unqualified	0.781	0.297	3.218	2.315 (1.645, 4.156)	< 0.001
**MAD**					
Qualified	–	–	–	1	–
Unqualified	0.618	0.362	2.855	1.865 (1.362, 3.673)	< 0.001

## Discussion

Xinjiang is located in the remote area of Northwest China. Compared with the southeast coastal area of China, its economic development and cultural development are lagging behind. Children are the hope of national development and social progress; therefore, the health status of infants and young children has become a very important public health concern^6^. Infancy is a key period of growth and development, which has a very important impact on healthy maturation^7^. A WHO report shows that malnutrition within 2 years after birth is the main cause of death in children under 5 years old^8^. Therefore, the results of this study show that the prevalence of malnutrition in Han infants is lower than that in other nationalities, especially in Uygur, which can reach 30.25%. This study used unconditional multi-factor logistic regression analysis to discover that Uygur, Hui, and other ethnic groups are more likely to suffer from malnutrition than the Han nationality; those who live in rural areas, are not mothers, did not breastfeed, had education below junior high school, and had a feeding behavior score of no more than 42, are independent risk factors. MDD, MMF, and MAD are not qualified to establish risk factors. All of the above shows that the nutritional health status of infants is affected by many risk factors. Therefore, this study aims to reveal the relevant risk factors, facilitate the formation of a scientific program, and provide a basis for the healthy growth of infants.

This study shows that Uygur and other ethnic minorities are mainly concentrated in southern Xinjiang, and mainly live in rural areas, while Han and Hui are mainly living in Northern Xinjiang. Compared with Northern Xinjiang, the economic development of Southern Xinjiang is relatively backward, and the concept of education is still improving. From the results of this study, it is also confirmed that the educational background of feeders is generally relatively low, lacking scientific feeding knowledge reserves and ideas. For this kind of phenomenon, we should first suggest local health institutions and educational institutions, which should be supported and given specific help. As a scientific research team, we will try our best to make scientific and reasonable relevant feeding knowledge brochures, so that more feeders can learn relevant knowledge.

The results of this study show that non-adherence to breastfeeding and the score of breastfeeding ≤ 42 are the independent risk factors of malnutrition in infants. It was found that the breastfeeding rate of Uygur, Hui, and other ethnic minorities is higher than that of the Han nationality. Most of the breastfeeding women of the Han nationality weaned their children in 5–6 months, while other ethnic groups maintained their traditional concept of long breastfeeding cycles. After communicating with the interviewees on the spot, it was found that many Han people think that milk powder can be used after feeding for several months. Owing to different ideas, the behavior and time of breastfeeding are different between different nationalities. However, most scholars believe that it is beneficial for children's growth and development to extend the period of breastfeeding properly. Through this phenomenon, we will further clarify the importance of breastfeeding in brochures and other media.

The qualified rate of MDD, MMF, and MAD plays an important role in the healthy growth of infants. The results of this study show that the qualified rate of MDD, MMF, and MAD in the Han nationality is higher than that of other nationalities, which is similar to the results of WAZ, HAZ and WHZ, and the following results are obtained from this aspect. The healthy growth of Han infants is better than that of other ethnic groups, which may be affected by the living environment, customs, social and economic development, education, and other factors of different ethnic groups.

In this survey and research, it was found that the nutritional health level of infants and young children in Xinjiang, China is relatively low, and nutritional levels within the Han nationality are better than that of other ethnic groups. Based on the results of this study, we can learn that making local residents accept more systematic and comprehensive health education and science popularization will play a positive role in the growth and development of local infants and young children. To a large extent, infants and young children are the future of a country or nation. Therefore, we will try to regularly send medical teams and local health institutions to take targeted interventions to improve the rational feeding methods of infants and young children and develop parental ideas, so as to promote the healthy growth of their children.

## Conclusions

Based on the analysis of the current situation and nutritional status of the survey results, the nutritional health level of infants in various regions and ethnic groups in Xinjiang is relatively low. At the same time, the differences in ethnic groups, urban and rural areas, North and South Xinjiang, and other factors are found. We found differences in educational level, customs, and habits among all ethnic groups, which leads to differences in the prevalence of infant malnutrition among all ethnic groups. Our research needs to be further strengthened and improved. Improving the scientific feeding rate is a huge project with various measures. In the future, we will get involved on the grass-roots level, give specific guidance, and follow-up on results.

## Supplementary information

Supplementary Information.

## Abbreviations

HAZ: Height for age Z score
MAD: Minimum acceptable diet
MDD: Minimum dietary diversity
MMF: Minimum meal frequency
WAZ: Weight for age Z score
WHZ: Weight for height Z score

## References

[1] Ahluwalia N, Herrick K, Paulose-Ram R, Clifford J (2014). Data needs for B-24 and beyond: NHANES data relevant for nutrition surveillance of infants and young children. Am. J. Clin. Nutr..

[2] Ana BP, Klein BP (2009). Impact of fortified blended food aid products on nutritional status of infants and young children in developing countries. Nutr. Rev..

[3] Ahmad I, Khalique N, Khalil S, Urfi MM (2017). Complementary feeding practices among children aged 6–23 months in Aligarh, Uttar Pradesh. J. Fam. Med. Primary Care.

[4] Qian-Qian LI, Qian L, Jun-Mei Y, Wang X (2018). Effects of different feeding patterns on the growth and development of infants with very/extremely low birth weight. Chin. J. Contemp. Pediatr..

[5] Mercedes DO, Adelheid WO, Elaine B, Cutberto G (2006). Comparison of the World Health Organization (WHO) Child Growth Standards and the National Center for Health Statistics/WHO international growth reference: implications for child health programmes. Public Health Nutr..

[6] Nita B, Mercedes DO (2007). The current status of international standards for child growth. Indian J. Med. Res..

[7] Madhur B, Rupali B, Kanika KBA (2015). cross sectional study on health status of infants in rural areas of Kamrup, Assam. Indian J. Community Health.

[8] Zong YD, Liu XH (2013). Interpretation of WHO Global Strategy for infant and young children feeding. Zhonghua Er Ke Za Zhi Chin. J. Pediatr..

