# Supplementary material for: Assessment of the nutritional status of 6–36-month-old infants in Xinjiang and analysis of the influencing factors
Source: Sci Rep. 2020 Dec 3;10:21031. doi: 10.1038/s41598-020-78042-6 (PMC7713047; doi:10.1038/s41598-020-78042-6)
Supplement: Supplementary file 1 — Supplementary Information. [file 41598_2020_78042_MOESM1_ESM.doc]

**Personal number： □ □ □ □**

**Questionnaire of infant feeding index and malnutrition composite index** Dear parents: Hello, thank you for your cooperation：

The purpose of this survey is to understand the status quo of feeding practice and physical development of infants and their feeders from 0 to 36 months in Xinjiang. Please fill in the form below truthfully. Your information is only for group analysis and will not be leaked out. Thank you for your support. The First Affiliated Hospital of Xinjiang Medical University

**A1 About infants**

1、Full Name：__________，date of birth ＿ ＿

2、Height__cm;weight;___ kg;Upper arm circumference____cm;Head circumference_____cm

3、Gender：①male ②female

4、Nation：①Han ②Uygur ③Kazakh ④Hui ⑤other________

5、household registration:①Urban②Rural Home address______________________

6、Primary caregivers： ①parents ②Grandparents ③Both ④other____________

7、Vaccination：①Yes ___________ ②No

8、Birth weight_______kg

9、Full term birth①Yes ②No

10、Mode of delivery①Natural childbirth ②caesarean birth

11、Production location：①hospital ②home

12、Regular physical examination for infants and young children：①42days ②3months ③6months ④9months ⑤1year ⑥1.5year ⑦2year ⑧2.5year ⑨3year

13、Is it the first child ： ①Yes ②No

14、If the child has ever had a serious illness?

①Yes How old are you sick ______ , What diseases do you have ______ ②No

**A2 About mothers of infants**

15、Age_______

16、Education： ①No school ②primary school ③junior middle school ④high school ⑤junior college ⑥Bachelor degree or above

17、Occupation： ①worker ②farmer ③cadre ④housework ⑤other

**A3 About fathers of infants**

18、Age________

19、Education： ①No school ②primary school ③junior middle school ④high school ⑤junior college ⑥Bachelor degree or above

20、Occupation： ①worker ②farmer ③cadre ④housework ⑤other

**A4 Family part of infants**

21、Number of children in the family： ①1 ②2 ③3 ④More than 4

22、Family income：①＞1500RMB / month ② 1500-2999RMB / month ③3000-5999RMB / month ④≥6000RMB / month

23、Family property（Multiple choices are available） ：

① Telephone ②mobile phone ③television ④Washing machine ⑤Refrigerator ⑥car ⑦Agricultural machinery ⑧computer

**B1 Feeding index**

24、The source of children's feeding knowledge（Multiple choices are available）：①Active access to feeding information ② Television broadcasting ③ newspapers and magazines ④ health guidance for medical staff ⑤ publicity materials ⑥ family members or neighbors ⑦mass media ⑧ pregnant women's school

25、How old is weaning cut off in infancy_____year____months，Reasons for weaning________

26、If not exclusive breastfeeding, which of the following would you prefer：

① Milk ② goat milk ③ milk powder ④ formula milk (Band＿＿＿）

⑤ Breast milk + milk ⑥ breast milk + milk + milk powder ⑦breast milk + milk + formula milk

27、Daily feeding times of complementary food in the past 24 hours：

① 0 times / day ② 0-1 times / day ③ 1 times / day ④ 1-2 times / day ⑤ 2-3 times / day ⑥ ≥ 3 times / day

28、Times of feeding complementary food between two meals per day in the past 24 hours：

① 0 times / day ② 0-1 times / day

29、Bottle feeding in the past 24 hours ①Yes ②No

30、The past 24 hours have been diverse：

Food types ① Vegetables (fruits) ② eggs ③ meat ④ fish and shrimp ⑤ beans and products ⑥ grains and potatoes

31、Food types：(Within the last 24 hours）

① Vegetables (fruits) ② eggs ③ meat ④ fish and shrimp ⑤ beans and products ⑥ milk and products ⑦ grains and potatoes

32、Number of days in the past week to eat all kinds of foods (including high calorie foods, Cereals, eggs, milk, meat, beans, vegetables and others)

① No intake; ② 1-3 days / week; ③ ≥ 4 days / week

33、High calorie food (butter, sweet tea, nang)：① Once / week ② 2 times / week ③ 3 times / week ④ 4 times / week

34、Bean products: ① less than 1 time / month; ② 1 - 4 times / week; ③ ≥ 5 times / week

35、Eggs: ① less than 1 time / month; ② 1 - 4 times / week; ③ ≥ 5 times / week

36、Meat: ① less than 1 time / month; ② 1 - 4 times / week; ③ ≥ 5 times / week

37、Fruits and vegetables: ① less than 1 time / month; ② 1 - 4 times / week; ③ ≥ 5 times / week

72、Cereals:：① less than 1 time / month; ② 1 - 4 times / week; ③ ≥ 5 times / week

73、Milk and dairy products：① less than 1 time / month; ② 1 - 4 times / week; ③ ≥ 5 times / week

74、Fish：① less than 1 time / month; ② 1 - 4 times / week; ③ ≥ 5 times / week

**B2、Time of first addition of complementary food（High calorie foods are not included）**

38、Starch：①2months ② 3months ③4months ④5months ⑤6months ⑥7months ⑦8months ⑧9months ⑨10months

39、Vegetables and fruits：①2months ② 3months ③4months ④5months ⑤6months ⑥7months ⑦8months ⑧9months ⑨10months

40、Eggs: ①2months ② 3months ③4months ④5months ⑤6months ⑥7months ⑦8months ⑧9months ⑨10months

41、Fish and liver：①2months ② 3months ③4months ④5months ⑤6months ⑥7months ⑦8months ⑧9months ⑨10months

42、Other meat：①2months ② 3months ③4months ④5months ⑤6months ⑥7months ⑦8months ⑧9months ⑨10months

43、Cod liver oil preparation：①2months ② 3months ③4months ④5months ⑤6months ⑥7months ⑦8months ⑧9months ⑨10months

44、tea with milk： ①2months ② 3months ③4months ④5months ⑤6months ⑥7months ⑦8months ⑧9months ⑨10months

**B3 (Fill in the following information for infants）**

45、Daily milk volume： ①100ml ②200ml ③300ml ④400ml ⑤≥500ml

46、Eggs intake

① One per day ② one every two days ③ one every three days ④ one every week ⑤ one occasionally

47、Daily intake of meat, poultry and fish：① < 15g / day ② 15-75g / day ③ ≥ 75g / day

48、Amount of salt added in making supplementary food for infants：① No salt is added; ② the taste of adults is the same; ③ the amount of salt added is obviously less than that of adults

49、Sugar content in making infant supplementary food：①Often ②occasionally ③ Never

**5**0、Drinking water or milk from a cup：①7months ②8months ③ 9months ④10months ⑤11months ⑥12months

**C1 Feeding behavior of feeders**

51、Prepare food for children alone:

①always ②often ③Sometimes ④rare ⑤never

52、Food preparation focuses on meeting the needs of age：

①always ②often ③Sometimes ④rare ⑤never

53、There is a fixed feeding position:

①always ②often ③Sometimes ④rare ⑤never

54、There are special tableware：

①always ②often ③Sometimes ④rare ⑤never

55、After feeding：

①always ②often ③Sometimes ④rare ⑤never

56、Play with toys / watch TV while eating：

①always ②often ③Sometimes ④rare ⑤never

57、Each meal lasts more than 30 minutes：

①always ②often ③Sometimes ④rare ⑤never

58、Face to face feeding：

①always ②often ③Sometimes ④rare ⑤never

59、Language encouragement in feeding：

①always ②often ③Sometimes ④rare ⑤never

60、Encourage independent eating：

①always ②often ③Sometimes ④rare ⑤never

61、No compulsive eating：

①always ②often ③Sometimes ④rare ⑤never

62、Pay attention to your child's hunger / satiety signals：

①always ②often ③Sometimes ④rare ⑤never

63、Describe the characteristics of the food and the feeding situation to the child verbally during feeding：

①always ②often ③Sometimes ④rare ⑤never

64、When the child refuses food and cries, the feeder feels depressed and angry：

①always ②often ③Sometimes ④rare ⑤never

65、Feed：

①Give sugar water or sweet fruit water every day ②Egg yolk added to formula ③Rice flour added to formula milk ④Each lactation time is less than 15 minutes

66、Feeding：

①Food other than milk has not been added in children aged over 12 months ② Food other than milk was given 4 months ago ③Only muddy food (non granulated food) was given to the aged ≥ 10 months ④Weaning for more than 10 months only eat all kinds of food

**C2 Knowledge and attitude of feeders**

67、Do you think：

①Breastfeeding is good ②Ordinary milk powder can be fed ③Good milk ④Good formula（Reasons for not using formula milk powder：_________________）

68、Do you think colostrum should

①discard ②Feed the baby

69、When do you think it is time to add complementary food

①4-6months ②Full 6 months ③Less than 4 months ④More than 7-8 months

70、When do you think weaning

①12months ②24months

**D**

71、The relationship between the filler and the child： ①father ②mother ③other________ contact information：_________

**Thank you for your cooperation!**
